# Supplementary material for: Dynalign II: common secondary structure prediction for RNA homologs with domain insertions
Source: Nucleic Acids Res. 2014 Nov 21;42(22):13939–48. doi: 10.1093/nar/gku1172 (PMC4267632; doi:10.1093/nar/gku1172)
Supplement: SUPPLEMENTARY DATA [file supp_gku1172_nar-02021-z-2014-File012.zip › manual/Text/MaxExpect.html]

RNAstructure Command Line Help: MaxExpect


|  |  |  |
| --- | --- | --- |
|  | RNAstructure Command Line Help MaxExpect | - Contents - Index |
| MaxExpect predicts the maximum expected accuracy structure, a structure that maximizes pair probabilities. USAGE: MaxExpect <input file> <ct file> [options] Required parameters: <input file> \| The name of a file containing input data. This input data can be in one of two formats: 1. Partition function save file (holds base pairing probability data for all pairs and can be generated using the partition interface). 2. Sequence file (holds raw sequence: .seq or .fasta). Note that lowercase nucleotides are forced single-stranded in structure prediction. Note that in order to use a squence file, the "sequence" flag must be specified (see "--sequence" below).; <ct file> \| The name of a CT file to which output will be written. Options which don't require added values: -d, -D, --DNA \| This flag only matters if the input file is a squence file and has been identified as such (see "--sequence" below). Specify that the sequence is DNA, and DNA parameters are to be used. Default is to use RNA parameters.; -h, -H, --help \| Display the usage details message.; --sequence \| Identify the input file format as a sequence file. Options which require added values: -g, -G, --gamma \| Specify the weight which should be placed on base pairs. Default is 1.0.; -p, -P, --percent \| Specify a maximum percent difference in folding free energy change in suboptimal structures. Note that suboptimal structures are generated until either the maximum percent free energy difference is reached or until the maximum number of structures is reached (below). Default is 50 percent (specified as 50, not 0.5).; -s, -S, --structures \| Specify a maximum number of structures. Note that suboptimal structures are generated until either the maximum number of structures are reached or the maximum percent difference is reached (above). Default is 1000 structures.; -w, -W, --window \| Specify a window size. Smaller windows (down to zero) will result in a larger set of structures that are similar. Larger windows result in fewer structures that are more different in predicted pairs. Default is 5. References: 1. Reuter, J.S. and Mathews, D.H. "RNAstructure: software for RNA secondary structure prediction and analysis." *BMC Bioinformatics*, 11:129. (2010). 2. Lu, Z.J., Gloor, J.W. and Mathews, D.H. "Improved RNA Secondary Structure Prediction by Maximizing Expected Pair Accuracy." *RNA*, 15:1805-1813. (2009). | | |
| Visit The Mathews Lab RNAstructure Page for updates and latest information. | | |
